# Corpectomy and spinal stabilization using a 3D‐printed spine model and custom jigs to address severe spinal deformities from T9‐11 and L2‐4 in a 6‐month‐old German shepherd puppy

**DOI:** 10.1002/ccr3.5229

**Published:** 2021-12-26

**Authors:** Charlotte G. Musser, Rebecca C. Windsor, Fred Wininger

**Affiliations:** ^1^ Wheat Ridge Animal Hospital Wheat Ridge Colorado USA; ^2^ Charlotte Animal Referral and Emergency Charlotte North Carolina USA

**Keywords:** congenital, malformation, spinal, surgery, vertebrae

## Abstract

This report describes surgical decompression and stabilization of 2 hemivertebrae in a German shepherd dog. Long‐term clinical and imaging outcomes are documented. Spinal cord decompression via corpectomy improved neurological function and intrinsic spinal cord changes on MRI. The dog improved to have minimal paraparesis and an active lifestyle.

## Graphical abstract

T2W sagittal preoperative image. Note the marked T2W hyperintensity from T6‐T8 and at L1 (white arrows) consistent with syringomyelia and marked spinal cord compression with dilation of the subarachnoid space at the T10 and L3 hemivertebrae sites (red arrows).

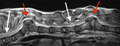

## 1 INTRODUCTION

Congenital vertebral malformations occur commonly in dogs and are most frequently reported in English and French bulldogs, Pug dogs, and Boston terriers.^1^, ^2^, ^3^ Reports of severe congenital malformations in large dogs are rare,^4^ and there are no reports of multiple severe congenital vertebral anomalies in a large breed dog.

During development, the vertebrae develop from the paraxial mesoderm surrounding the notochord, which divides into paired somites, each of which develops into a sclerotome (which gives rise to the bony components of the axial skeleton) and a dermomyotome (which gives rise to the musculature and dermis).^5^, ^6^ Cells of the sclerotome migrate to surround the notochord, and then the neural tube.^5^, ^7^ At this point, the sclerotome divides into a cranial and caudal aspect, between which the intervertebral disc forms from the notochord and the caudal sclerotome. The then‐separated cranial and caudal aspects of the sclerotome fuse with the cranial and caudal aspects of the adjacent sclerotome, forming the centrum of the vertebrae.^5^, ^8^, ^9^ Failures of formation of the vertebral body result in hemivertebra, wedge‐shaped vertebra, and “butterfly” vertebra.^3^

While many dogs have no clinical signs associated with these abnormalities, others can have severe neurologic dysfunction including paraparesis, ataxia, and incontinence.^10^, ^11^ Signs may be chronic or have an acute onset or progression.^12^, ^13^ Medical and surgical management has been described with no consensus as to the best practice^1^ but reported surgical cases have generally good outcomes.^10^, ^11^ In a group of small dogs with congenital thoracic vertebral body malformations, non‐surgical management resulted in neurological deterioration in all dogs.^1^ Surgical techniques include stabilization with or without spinal cord decompression^4^, ^10^, ^11^, ^14^ and decompression without stabilization.^15^ Intraoperative pin placement in areas of severe kyphosis or scoliosis can be technically challenging. In human neurosurgery, rapid prototyping may reduce surgery times and improve intraoperative safety in correction of malformations.^16^, ^17^, ^18^

## 2 CASE HISTORY

**FIGURE 1:**
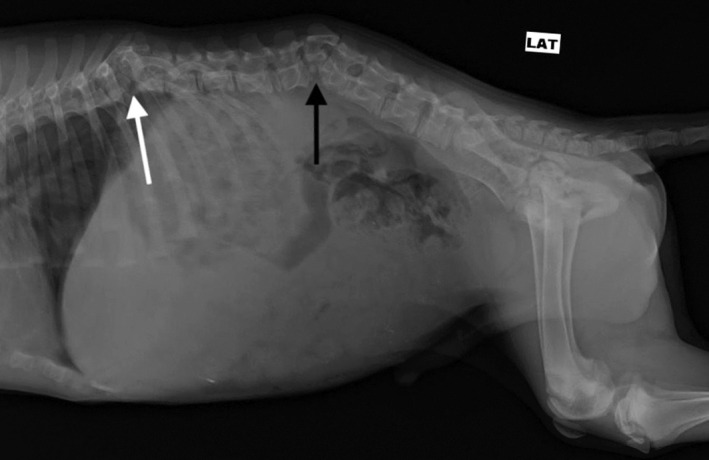
Lateral radiograph of vertebral anomalies taken at 3 months of age. White arrow pointing to the T10 hemivertebrae. Black arrow pointing to the L3 vertebrae

A recently rescued, 3‐month‐old male German shepherd dog was presented to a general practice veterinary clinic with a 2‐week history of mildly progressive paraparesis, with prior history being unknown. Radiographs showed dorsal hemivertebrae at T10 and L3 causing marked kyphosis (Figure 1). He was then presented to a veterinary specialty hospital, where on initial examination he was bright alert and responsive with normal cranial nerves. He was minimally ambulatory with severe pelvic limb paresis and proprioceptive ataxia. Pelvic limb paw placement was absent in the pelvic limbs and normal in the thoracic limbs. Thoracic and pelvic limb spinal reflexes were normal. Cutaneous trunci reflex was difficult to elicit. Urinary and fecal function was reportedly normal, and rectal tone was normal. Mild diffuse spinal pain was present on palpation. His neuroanatomic localization was a T3‐L3 myelopathy. Given the severity of the congenital vertebral malformation on radiographs, there was concern for compressive myelopathy. Infectious/inflammatory processes and other congenital malformations were also considered.

## 3 DIAGNOSTICS AND TREATMENT

### 3.1 Imaging

**FIGURE 2:**
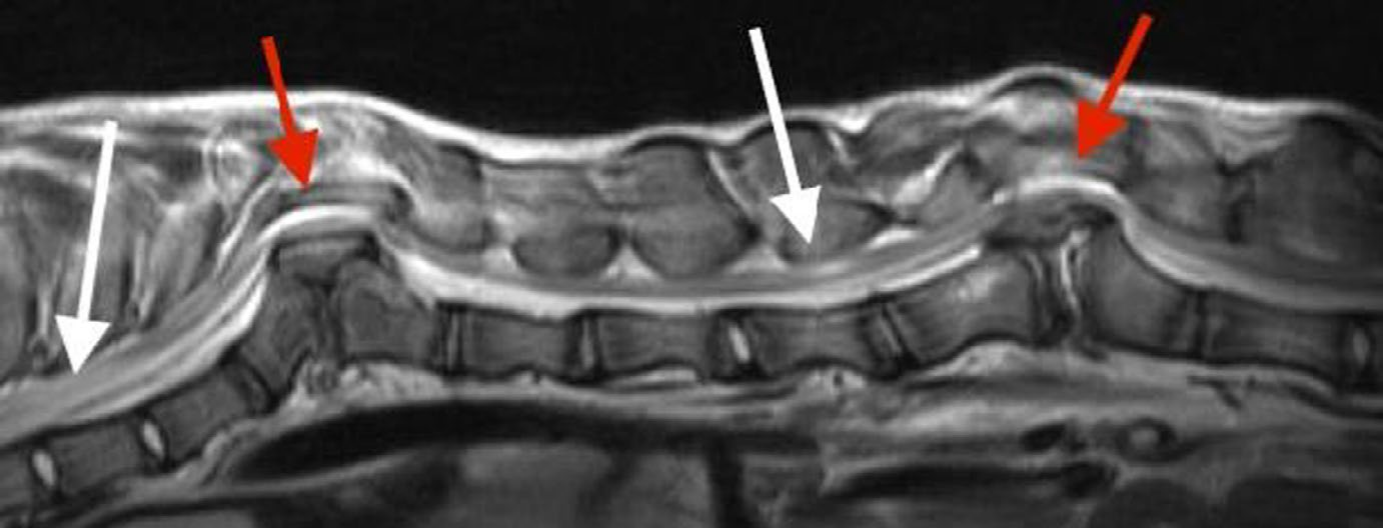
T2W sagittal preoperative image. Note the marked T2W hyperintensity from T6‐T8 and at L1 (white arrows) and marked spinal cord compression at the T10 and L3 hemivertebrae sites (red arrows)

**FIGURE 3:**
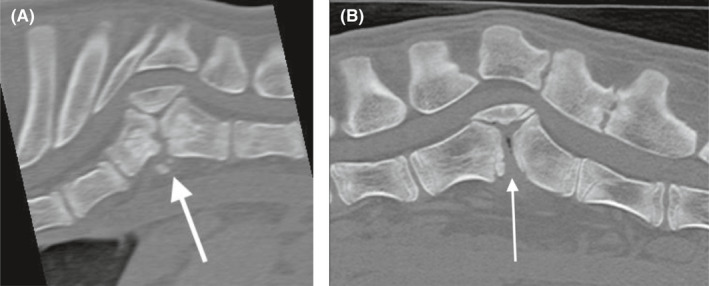
Preoperative CT image of the thoracic (A) and lumbar (B) spinal malformations. White arrows pointing to the sharp angle between T9, T10 (hemivertebrae), and T11 and between L2, L3 (hemivertebrae), and L4

Images were obtained using a 1.5 Tesla magnetic resonance imaging (MRI) (Siemens MAGNETOM Symphony) and 64‐slice helical computed tomography (CT) scanner (Toshiba Aquilion 64) when the dog was approximately 6 months old. Standard spin‐echo T2‐weighted (T2W) sagittal and transverse and dorsal STIR sequences were obtained through the thoracolumbar spine. Dorsal hemivertebrae were present at T10 and L3 (Figure 2). At T10, there was severe kyphosis and mild scoliosis, with severe spinal cord compression from the T9 to T11 vertebral bodies. At the T9‐10 and T10‐T11 intervertebral disc spaces, the spinal canal was approximately 50% of its normal diameter. At L3, there was moderate kyphosis and scoliosis. At the L2‐3 and L3‐4 intervertebral disc spaces, the spinal canal was approximately 70% of its normal diameter. There was a large T2W hyperintensity within the center of the spinal cord consistent with a syrinx formation cranial to T9. There was moderate‐to‐severe dilation of the subarachnoid space dorsal to T10 consistent with early subarachnoid diverticulum formation, and severe dilation of the dorsal subarachnoid space at L3. There was moderate‐to‐severe T2W intramedullary hyperintensity consistent with a potential focal syrinx from T6‐8 and L1. Computed tomography (64‐slice helical CT, 1 mm, bone algorithm) images were obtained to better visualize the vertebrae for pre‐surgical planning (Figure 3A,B). Preoperative Cobb angles measured using the CT images were 45° from T9‐11 and 35.6° from L2‐4.^1^, ^19^

### 3.2 3D modeling

**FIGURE 4:**
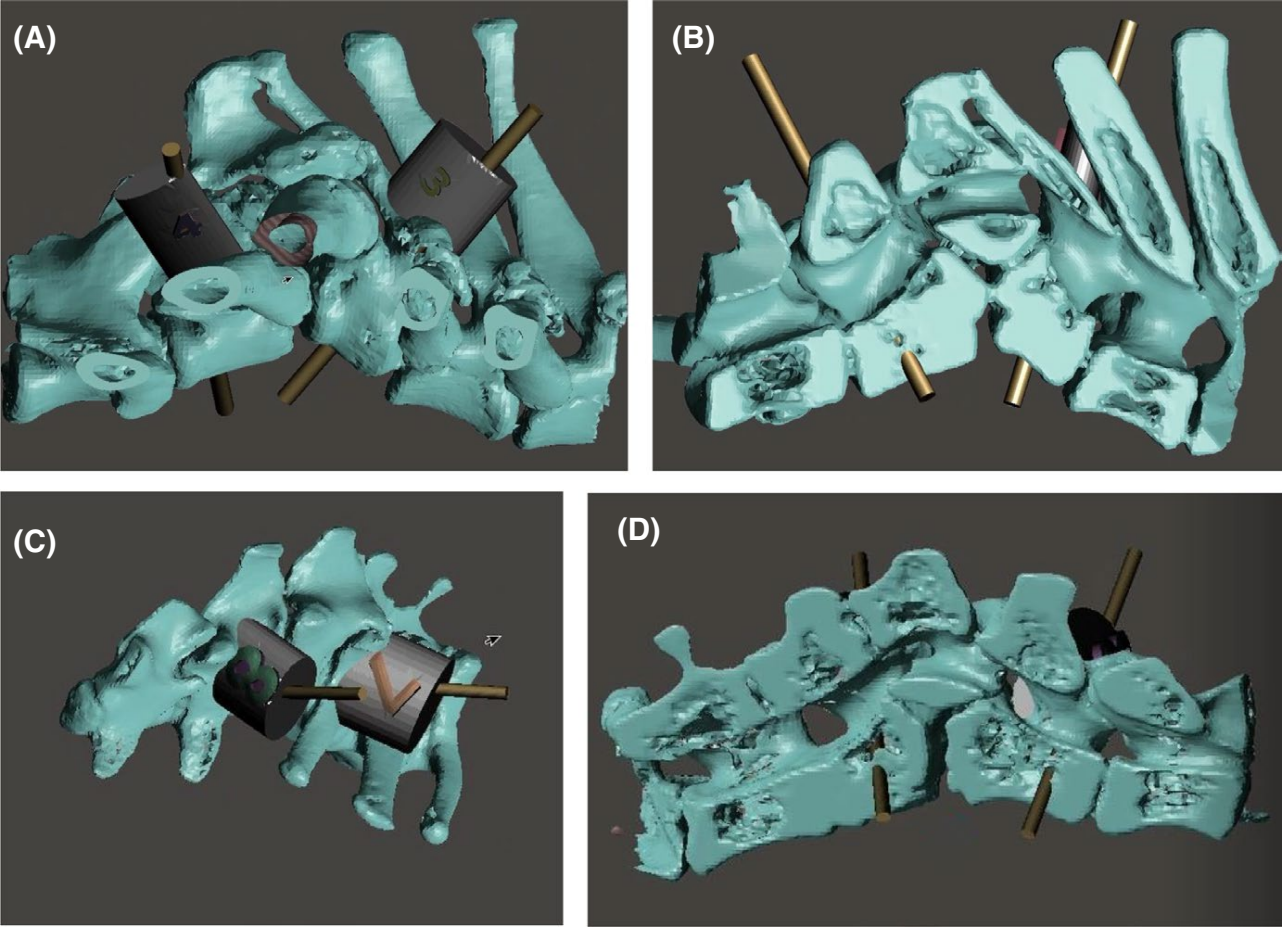
Computerized model of spinal malformation with examples of lateral and sagittal sectional imaging to demonstrate jig/drill guide placement on the T9, T11 (A, B), and L2, L4 (C, D)

Computed tomography scans were imported into HOROS software (V3.3.5). Thresholding was performed selecting for voxels with Hounsfield units between 800 and 3000. A 3D volume rendering was created, and manual subtraction was performed removing extraneous tissues outside the vertebral segments of interest. The surface rendering was compiled and exported as an STL file. The STL file was imported into the Meshmixer software (v 3.5.474). Extraneous “shells” were separated and deleted. Cylindrical trajectories were made through the individual vertebrae with a diameter of 2.7 mm. The trajectories fit three criteria. (1) They had an entry point on the lamina of the vertebrae. (2) They crossed midline with an exit point on the contralateral vertebral body. (3) Their corridor was wide enough such that there was a 2 mm gap between them and both inner and outer laminar cortices (Figure 4A–D).

Once trajectories were made, interdigitating footplates were created in the lamina to encase the trajectories. The footplates incorporated the novel surfaces of the caudal articular process and spinous process to ensure they had a unique native fit. Those selected surfaces were extruded to a depth of 5mm. Through boolean operations, the trajectories were subtracted from the footplates to accommodate a drill bit and complete the jigs.

**FIGURE 5:**
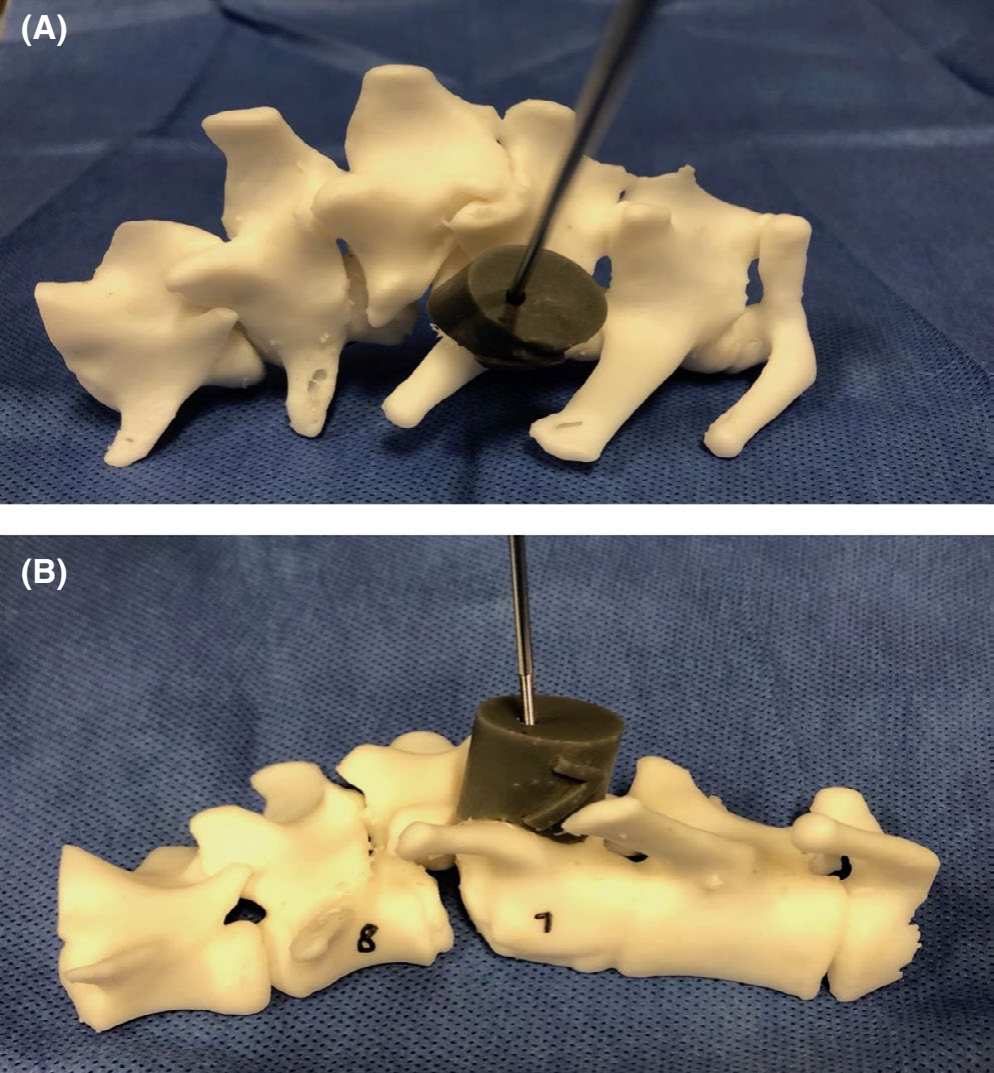
Print of the lumbar (A, B) spine showing custom drill guides that were placed on the vertebrae intraoperatively

The jig (drill guide) prints were exported to the Preform G slicer software (v3.0.1). The proprietary software automatically chose the ideal printing orientation and support strategy. The jig prints were modeled at 200 micron resolution in the Formlabs proprietary Dental SG resin. The resin is a photo‐activated polymer that hardens when illuminated by a 405 nm wavelength laser in the Form 2 stereolithography printer (Figure 5A,B).

After printing was complete, the prints were placed in a 90% isopropyl alcohol bath for 12 min. The supports were removed, and the print was soaked in the bath for another 12 min. The print was placed in a 405 nm LED curing chamber for 1 h.

### 3.3 Surgical technique

**FIGURE 6:**
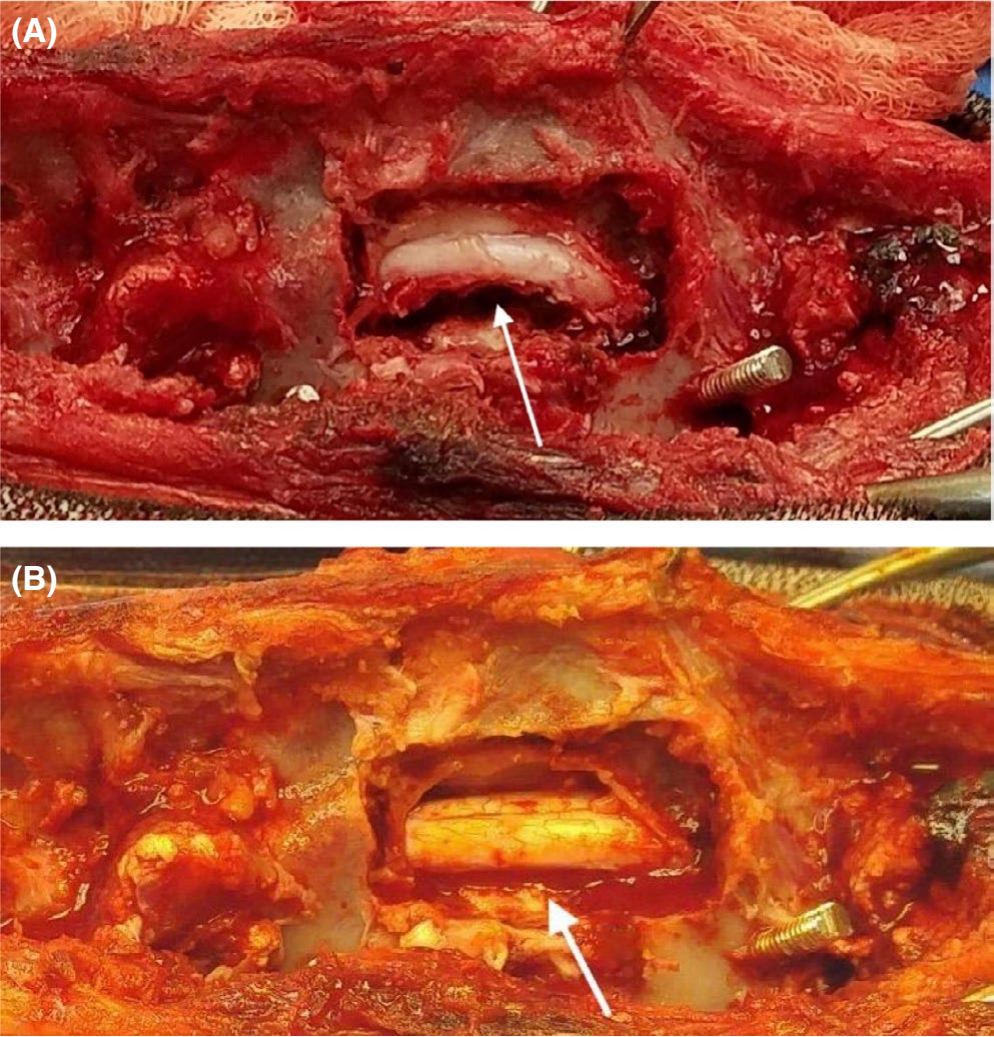
Image of the lumbar spinal cord following L2‐4 hemilaminectomy and prior to L3 corpectomy (A) and following completed L3 corpectomy (B). White arrows show marked spinal cord compression prior to removal of the dorsal shelf of bone from the vertebrectomy (A) compared to decompression post‐vertebrectomy (B)

The spine print was sterilized with ethylene oxide gas for 12 h and used intraoperatively for reference. The epaxial muscles were dissected bilaterally from T8‐L5 to expose all articular processes using a standard approach. Eight jig prints (labeled 1–8 corresponding to the left and right sides of T9, T11, L2, and L4) were placed onto the corresponding vertebral bodies where they were perfectly contoured to the vertebrae with a snug fit to allow for accurate pin trajectory. A 2.5 mm drill bit was used to predrill all pin holes, using the hole within the jig print as a guide. The jigs were all then removed prior to placement of positive profile spinal pins. Bilateral 2.4/3.2 mm positive profile pins were placed into the vertebral bodies of T9 and T11 and bilateral 2.8/3.5 mm positive profile extra‐long thread pins were placed into the vertebral bodies of L2 and L4. After the pins were placed and cut, hemilaminectomies were made on the left side from the caudal aspect of T9 through the cranial aspect of T11 and from the caudal aspect of L2 to the cranial aspect of L4 based on surgeon preference to approach from the left side. Lateral corpectomies were made on the left side to remove the entire T10 and L3 vertebral bodies, the dorsocaudal vertebral body of T9 and L2, and the dorsocranial vertebral body of T11 and L4. The spinal cord was deviated dorsally and compressed at both locations prior to the corpectomy (Figure 6A) and appeared flat in the vertebral canal post‐corpectomy (Figure 6B). The spinal cord was severely adhered in multiple locations laterally and ventrally at both T10 and L3, necessitating some gentle dissection with a #11 blade. The pins were fixed using polymethylmethacrylate bars on each side. Routine closure of the fascial, subcutaneous, and subcuticular layers was performed with adequate apposition.

### 3.4 Outcome and follow‐up

A CT scan immediately postoperatively showed accurate pin placement within the vertebral bodies at the desired angles without encroachment into the spinal canal.

At 48 h postoperatively, the dog was non‐ambulatory severely paraparetic but was able to stand with support. At 1 week postoperatively, he was non‐ambulatory moderately paraparetic and able to walk with sling support.

Neurologic examinations were performed by a neurologist at 2 weeks postoperatively and every 4 weeks for several months, and the dog was in rehabilitation therapy with a certified canine rehabilitation therapist at least once monthly for 17 months postoperatively. He showed gradual neurologic improvement. A CT scan was repeated at 3.5 months to evaluate the surgical sites. There was mild proliferation of the lamina on the right side from T9‐11 and L2‐4 with no spinal cord compression. At 7 months postoperatively, he was able to run but had mild paresis and delayed paw placement of the right pelvic limb. At 1 year postoperatively, he had normal paw placement with no pelvic limb paresis and was able to run and jump. He had intermittent right pelvic limb paresis over the next year mainly with long walks and more vigorous activity.

At 17 months postoperatively, the dog developed a colonic torsion and had surgery for derotation, colopexy, and prophylactic gastropexy. Review of the previous MRI and CT imaging showed no anatomic changes to indicate a predisposition to colonic torsion but imaging had been focused on the spine at that time. He was presented for swelling over the lumbar spine 10 months later. A draining tract with purulent discharge was present on the left dorsolateral cranial abdomen adjacent to the L2‐L4 implants. Oral amoxicillin/clavulanic acid was started, and draining tract material was submitted for aerobic and anaerobic culture, growing a multidrug‐resistant *Staphylococcus pseudintermedius* (MRSP). Amoxicillin/clavulanic acid was discontinued, and enrofloxacin (10 mg/kg orally once daily) was started based on sensitivity results. Topical mupirocin was started 1 month later. Both antibiotics were continued for 3 months. The left‐sided draining tract gradually resolved, but another draining tract developed on the caudal aspect of the right dorsolateral thorax. It was then decided to remove the implants from both surgical sites.

A CT scan prior to implant removal showed adequate bony fusion at the corpectomy sites and no lytic changes in the disc spaces or bone indicating discospondylitis or osteomyelitis. Following implant removal, enrofloxacin was continued and clindamycin (20 mg/kg orally twice daily) was started based on culture results. Within several weeks of implant removal, the dog developed left pelvic limb lameness, which improved when a tapering anti‐inflammatory course of oral prednisone was started. He was ambulatory with minimal paraparesis and no spinal pain or lameness within 2 weeks of implant removal.

**FIGURE 7:**
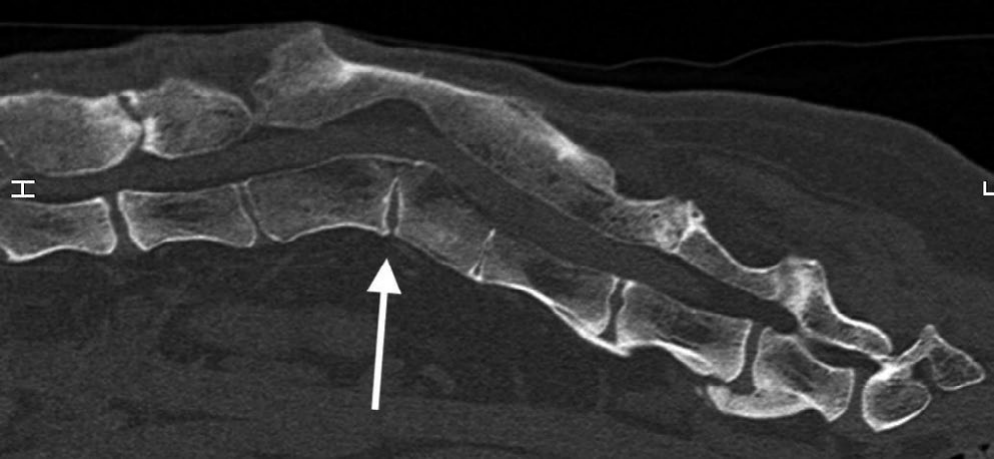
Sagittal CT image from the lumbar spine acquired 34 months postoperatively. White arrow demonstrates the smooth margin and fusion at the vertebrectomy site

**FIGURE 8:**
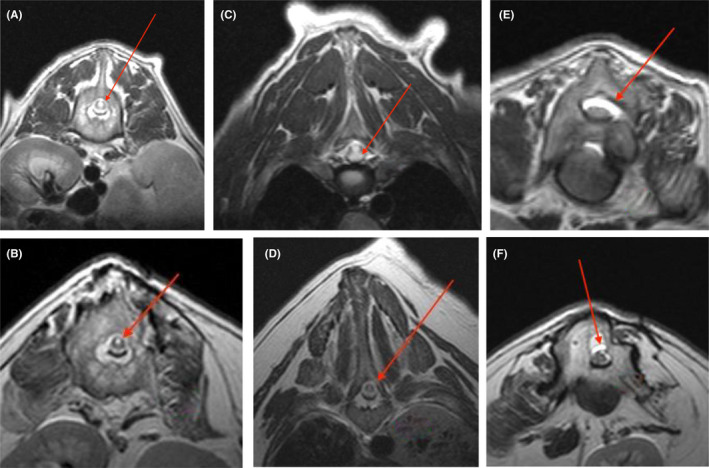
(A–F) T2 transverse MRI acquired at L1 preoperatively (A) and at 34 months postoperatively (PO) (B), T6‐7 preoperatively (C) and 34 months PO (D), and L3 preoperatively (E) and 34 months PO (F). Note the marked hyperintensity consistent with syrinx formation demonstrated by the red arrows in the preoperative transverse images at L1 (A) and T6‐7 (C) compared to the postoperative images at L1 (B) and T6‐7 (D) and the marked subarachnoid space dilation in the preoperative image at L3 (E) compared to the postoperative image (F)

Recheck MRI/CT was performed 34 months following the initial surgery (3 months after implant removal). The CT scan showed significant bony fusion ventrally and mild change in the Cobb angle with a postoperative T9‐11 Cobb angle of 61° (vs. 45°), and an L2‐4 of 49° (vs. 35.6°) (Figure 7). Comparison of transverse T2‐weighted images noted in Figure 8A (preoperative) and 8B (34 months postoperative) shows significant improvement in the degree of central canal dilation (suspect syringomyelia) at L1. Marked improvement is also noted when comparing the central canal dilation at the level of T6‐7 in images 8C (preoperative) and 8D (34 months postoperative). Marked dilation of the subarachnoid space at the level of L3 noted in transverse T2‐weighted preoperative imaging (8E) appears improved in postoperative imaging at 34 months (8F).

## 4 DISCUSSION

To the authors’ knowledge, this is the first report to describe spinal decompression and stabilization at multiple sites in a large breed dog with a 3‐year follow‐up and repeated MRI and CT imaging. Based on the severity and progression of paraparesis and the severe spinal cord compression and intrinsic changes, surgical intervention was elected despite the dog's skeletal immaturity. The dog's clinical signs improved, as did the extrinsic and intrinsic structural abnormalities noted on CT and MRI. The use of a 3D‐printed spine model with custom jigs was helpful in the planning and execution of surgery. Although there were no publications on the use of intraoperative 3D‐printed drill guides at the time the surgery was performed, very recent publications at the time of this publication highlight the excellent accuracy that can be achieved using jig prints to guide pin placement.^20^, ^21^, ^22^

In humans, early surgical correction of congenital kyphosis is generally recommended^24^ as the angle of kyphosis may increase during growth, correction of the abnormal angle may be easier,^24^ and progressive neurologic deterioration may be avoided.^25^ Multiple surgical procedures are used depending on the severity, location, and type of malformation, and the age of the patient. Reported techniques include hemiepiphysiodesis (tethering of growth on one side of the spine to allow for compensatory growth of the contralateral side of the spine), spinal fusion with or without implant placement, fusionless instrumentation systems, hemivertebra excision, vertebral column resection,^23^ and closing wedge osteotomy.^25^ Reported techniques in dogs are varied. Non‐surgical management was associated with a universally poor outcome in a review of 13 small dogs with congenital thoracic vertebral malformation, with all dogs ultimately having significant neurological decline.^14^ Although spinal stabilization is not always considered necessary when addressing a vertebral malformation, in this case, there was concern for vertebral instability given the large corpectomies at two locations which were relatively distant from each other. Vertebral bone proliferation was also a concern given the dog's age, but postoperative CT scans did not show encroachment of bone into the spinal canal. The ventral bridging spondylosis provided additional vertebral fusion and suggested that removing the implants 2.5 years later should not cause significant destabilization. The Cobb kyphosis angles of both deformities were improved postoperatively, but as the improvements were mild we suspect the spinal cord decompression contributed more to the improvement of the intrinsic spinal cord changes.

Implant removal provided the opportunity for repeat MRI without implant‐associated artifact to document improvement of the intrinsic spinal cord changes. Because this is often not possible in similar cases, it is difficult to assess whether clinical improvement is a result of improved intrinsic spinal cord changes, reduced spinal cord compression, or if gait improves via other mechanisms (i.e., recruitment of other neural input, rehabilitation therapy which shows promise but has not yet been adequately evaluated for this specific type of chronic myelopathy).

Hemilaminectomy/corpectomy and stabilization with guidance of a printed spine model and custom printed jigs were effective in improving the neurological abnormalities and intrinsic spinal cord changes in this dog.

## CONFLICT OF INTEREST

The authors declare no conflict of interest related to this report.

## AUTHOR CONTRIBUTIONS

RCW: Attending veterinarian and surgeon, manuscript drafting, and review. CGM: Manuscript drafting and review. FW: 3D modeling and printing, and manuscript drafting.

## ETHICAL APPROVAL

Client consent was obtained for this publication.

## CONSENT

Written consent was obtained from the owner for publication of this manuscript.

## Data Availability

Data sharing is not applicable to this article as no new data were created or analyzed in this study.
